# Constitutive activation of the ERK pathway in melanoma and skin melanocytes in Grey horses

**DOI:** 10.1186/1471-2407-14-857

**Published:** 2014-11-21

**Authors:** Lin Jiang, Cécile Campagne, Elisabeth Sundström, Pedro Sousa, Saima Imran, Monika Seltenhammer, Gerli Pielberg, Mats J Olsson, Giorgia Egidy, Leif Andersson, Anna Golovko

**Affiliations:** Science for Life Laboratory, Department of Medical Biochemistry and Microbiology, Uppsala University, Box 582, SE-751 23 Uppsala, Sweden; INRA, Unité Mixte de Recherche 955, Génétique fonctionnelle et médicale, 7, avenue du Général de Gaulle, 94704 Maisons-Alfort, Cedex, France; Ecole Nationale Vétérinaire d’Alfort, Maisons-Alfort, France; Department of Forensic Sciences, Medical University of Vienna, Sensengasse 2, 1090 Vienna, Austria; Department of Medical Sciences, Dermatology and Venereology, Uppsala University Hospital, SE-751 85 Uppsala, Sweden; ParkCell AB, Uppsala Science Park, Uppsala, Sweden; Institut Pasteur, USC INRA Génétique fonctionnelle de la Souris, 25 rue du Docteur Roux, 75724 Paris, Cedex, France; Unité de Recherche Associée 2578, Centre National de la Recherche Scientifique, Paris, France; Department of Animal Breeding and Genetics, Swedish University of Agricultural Sciences, Box 597, SE-751 24 Uppsala, Sweden; Institute of Beijing Animal Science and Veterinary, Chinese Academy of Agricultural Science, Beijing, 100194 China

**Keywords:** Melanoma, Grey horse, ERK pathway, STX17, Melanocytes

## Abstract

**Background:**

Constitutive activation of the ERK pathway, occurring in the vast majority of melanocytic neoplasms, has a pivotal role in melanoma development. Different mechanisms underlie this activation in different tumour settings. The Grey phenotype in horses, caused by a 4.6 kb duplication in intron 6 of *Syntaxin 17* (*STX17*), is associated with a very high incidence of cutaneous melanoma, but the molecular mechanism behind the melanomagenesis remains unknown. Here, we investigated the involvement of the ERK pathway in melanoma development in Grey horses.

**Methods:**

Grey horse melanoma tumours, cell lines and normal skin melanocytes were analyzed with help of indirect immunofluorescence and immunoblotting for the expression of phospho-ERK1/2 in comparison to that in non-grey horse and human counterparts. The mutational status of *BRAF*, *RAS*, *GNAQ*, *GNA11* and *KIT* genes in Grey horse melanomas was determined by direct sequencing. The effect of RAS, RAF and PI3K/AKT pathways on the activation of the ERK signaling in Grey horse melanoma cells was investigated with help of specific inhibitors and immunoblotting. Individual roles of RAF and RAS kinases on the ERK activation were examined using si-RNA based approach and immunoblotting.

**Results:**

We found that the ERK pathway is constitutively activated in Grey horse melanoma tumours and cell lines in the absence of somatic activating mutations in *BRAF*, *RAS*, *GNAQ*, *GNA11* and *KIT* genes or alterations in the expression of the main components of the pathway. The pathway is mitogenic and is mediated by BRAF, CRAF and KRAS kinases. Importantly, we found high activation of the ERK pathway also in epidermal melanocytes, suggesting a general predisposition to melanomagenesis in these horses.

**Conclusions:**

These findings demonstrate that the presence of the intronic 4.6 kb duplication in *STX17* is strongly associated with constitutive activation of the ERK pathway in melanocytic cells in Grey horses in the absence of somatic mutations commonly linked to the activation of this pathway during melanomagenesis. These findings are consistent with the universal importance of the ERK pathway in melanomagenesis and may have valuable implications for human melanoma research.

**Electronic supplementary material:**

The online version of this article (doi:10.1186/1471-2407-14-857) contains supplementary material, which is available to authorized users.

## Background

Deregulation of the extracellular signal-regulated kinase (ERK) pathway through hyperactivation is strongly associated with melanomagenesis [1, 2], with constitutively activated ERK1/2 being found in the majority of melanocytic neoplasms [3]. However, it appears that the underlying mechanisms for the ERK activation differ between different entities. While the most common cause for ERK activation in human cutaneous melanoma is the presence of somatic mutations in BRAF and RAS kinases [4], these mutations are nearly absent in human uveal melanoma, where activation of the pathway has been linked to somatic mutations in closely related GTPases GNAQ and GNA11 in 83% of the cases [5]. These mutations are also present in 63.2% of blue nevi [5]. Activating mutations in and/or gene copy number increases of a receptor tyrosine kinase KIT, found in 39% of mucosal and 36% of acral melanoma [6], are a plausible cause of the ERK pathway activation in these tumour cells [7, 8]. Examples of other, less common, mechanisms underlying hyperactivation of the ERK pathway in melanocytic neoplasms include activating mutations in MEK kinases [9], overexpression of wild-type BRAF [10] and decreased expression of negative regulators of the pathway [11, 12].

Grey horses exhibit a fascinating pigment cell disorder phenotype manifested by gradual loss of coat pigmentation, vitiligo-like skin depigmentation and a high incidence of melanoma. It is estimated that ~80% of Grey horses older than 15 years have melanomas, while this is a rare condition in horses with other coat colors [13]. The primary tumours arise in the dermis of the glabrous skin under the tail, in the perianal and genital regions, lips and eyelids, but could also occur internally [13, 14]. Although most of the melanomas have a long initially benign growth period, up to 66% of these tumours may become malignant with metastases formation in other organs [15]. Despite the unusual clinical behaviour, the Grey horse melanomas (GHM) share common features with certain human cutaneous melanomas and malignant blue nevi, suggesting similarities in pathogenesis [16].

We have previously demonstrated that the causative mutation for the Grey horse phenotype encompassing the dramatically increased risk of melanoma development is a 4.6 kb duplication in intron 6 of *Syntaxin 17* (*STX17*) ([17]; referred to as *Grey* mutation thereafter). This dominant mutation constitutes a *cis*-acting regulatory mutation that upregulates the expression of both *STX17* and the neighboring gene *NR4A3* encoding Nuclear Receptor subfamily 4, group A, member 3. It is still an open question if upregulation of *STX17* or *NR4A3* expression is crucial, or if both events are required for the phenotypic effects associated with Grey phenotypes. We have recently demonstrated that the duplicated region contains a weak melanocyte-specific enhancer that becomes a strong enhancer when duplicated [18]. The tissue specificity is explained by the presence of two perfect binding sites for MITF (microphthalmia-associated transcription factor) within the duplicated sequence. This interpretation is strongly supported by results from transgenic zebrafish where the horse duplicated sequence could drive melanocyte-specific reporter expression and this activity was inhibited by silencing MITF using morpoholinos [18]. Furthermore, we have observed a positive correlation between the copy number of the *Grey* mutation and the melanoma progression, suggesting that the mutation might constitute a melanoma-driving element [19]. While the causative genetic link between the *Grey* mutation and development of Grey horse melanoma is well established, the molecular mechanism behind this link remains uncharacterized as well as it is not known whether additional somatic mutations are required for tumourigenesis.

Given the importance of the ERK pathway in melanomagenesis, we assessed its involvement in melanoma development in Grey horses. We found that the ERK pathway is constitutively activated in Grey horse melanoma tumours and cells in the absence of somatic oncogenic mutations in *BRAF*, *RAS*, *GNAQ*, *GNA11* and *KIT* that are associated with activation of this pathway in the majority of human melanocytic tumours. This increased ERK signaling is growth promoting and proceeds via B-, CRAF and KRAS kinases. Importantly, the ERK pathway was found to be highly activated in all epidermal melanocytes, suggesting a general predisposition to melanomagenesis in these horses.

## Methods

### Cell cultures and drug treatments

The human BL [20], Mel-Ho [21] and M5 [22] and horse HoMel-L1 and HoMel-A1 [21] melanoma cell lines were cultured in RPMI-1640 supplemented with 10% fetal bovine serum, 2 mm L-glutamine, 100 units/ml penicillin and 100 μg/ml streptomycin at 37°C and 5% CO_2_. The horse cell lines were derived from melanoma tumours excised as part of a treatment procedure at the Federal stud Piber veterinary clinic (Köflach, Austria) and therefore their establishment did not require ethics committee approval. For the drug treatment assays, U0126, LY294002 (Cell Signaling Technology, MA, USA) and L779450 (Calbiochem, Darmstadt, Germany) were dissolved in DMSO and added to the culture medium at final DMSO concentration of 0.1%. Cells were seeded in triplicates and the drug effect on cell growth was measured by Alamar Blue assay (Invitrogen AB, Carlsbad, CA, USA) after three days of culture. DMSO-treated cells served as control.

### Analysis of BRAF, RAS, GNAQ, GNA11 and KIT mutations

DNA was prepared using the DNeasy Blood & Tissue kit (Qiagen, Valencia, CA, USA). Exons 11 and 15 of *BRAF* and exons 1–6 of *NRAS* were sequenced in the human and horse cell lines and melanomas. In addition, exon 1 and 2 of *HRAS*, exons 1–3 of *KRAS*, and exon 5 of *GNAQ* were sequenced in Grey horse melanoma cell lines and tumours. The human amplicons were obtained as described by [4]. The primers and PCR conditions used to obtain the horse amplicons are given in the Additional file 1: Supplementary Methods.

### Western blot

Cells were lysed in a buffer containing 50 mM Tris (pH 7.5), 100 mM NaCl, 1 mM EDTA, 10% glycerol, 20 mM sodium fluoride, 2.5 mM sodium pyrophosphate, 1 mM sodium orthovanadate and 0.5% Triton X-100 with a protease- and phosphatase-inhibitor cocktails (Roche Diagnostics, Mannheim, Germany). Immunoblotting was performed with the following primary antibodies: rabbit polyclonal anti-ERK1/2 (C-16), anti-MEK1/2 (12-B), anti-BRAF (C-19), anti-NRAS (C-20), anti-SPROUTY2 (H-120), mouse monoclonal anti-α-tubulin (10D8; Santa Cruz), rabbit monoclonal anti-P-ERK1/2 (D13.14.4E XP; Thr^202^/Tyr^204^) and rabbit polyclonal anti-RKIP (# 4742; Cell Signaling).

### Tissue immunofluorescence

Paraffin-embedded Grey horse primary melanoma tumours (n = 17) and skin biopsies (n = 7) were obtained from In Histo veterinary pathology laboratory (Korneuburg, Austria) and Federal stud Piber veterinary clinic (Köflach, Austria), respectively. Analogous preparations of non-Grey horse primary melanomas (n = 12) and skin biopsies (n = 5) were obtained from IDEXX veterinary pathology laboratory (Alfortville, France) and Alfort School of Veterinary Medicine (Maisons-Alfort, France), respectively. The age of horses used for sampling ranged from 4 to 18 years. Although all collected samples underwent the same standard fixation/embedding procedure, we included additional samples of non-Grey skins (n = 7) from Köflach, Austria and melanoma (n = 2) and skins from Grey (n = 2) and non-grey (n = 2) horses from University Animal Hospital (SLU, Uppsala, Sweden) in order to rule out potential differences between the sampling’s sources and fixation proceedures. All the tissue samples used (both tumour and skin) were not obtained specifically for this study, but collected as part of a treatment procedure and/or for diagnostic purposes, and therefore this research meets the ethical standards for this kind of experimentation. Deparaffinised 5 μm sections were incubated at 4°C overnight with the following primary antibodies: mouse monoclonal anti-MITF from Invitrogen as melanocyte marker, rabbit monoclonal anti-P-ERK1/2 and rabbit polyclonal anti-ERK1/2 (the same as for the Western blot); followed by the respective fluorescent AlexaFluor-488 and AlexaFluor-555 secondary antibodies (Invitrogen).

### Image acquisition

Tissue immunolabelling experiments were performed using the same samples in different experiments to get comparable controls. Acquisition time was identical for the skin and melanoma series for each antibody. Carl Zeiss ApoTome microscope (Carl Zeiss, GmbH, Jena, Germany) 0.7 μm optical sections were processed with Zeiss-Axiovision program. Cultured cells confocal images were acquired using a Carl Zeiss LSM 510 Meta confocal laser scanning microscope and an Apochromat 63× oil objective with NA 1.4.

### Quantification of the immunofluorescence signal

AxioVision .zvi images were analyzed by counting the number of MITF positive cells in one optical image of the z stack together with the number of these cells also positive for P-ERK1/2 or ERK1/2. Two 40× fields per sample were quantified. Samples were analyzed blindly by two authors (CC, GE). Statistical differences between the means of Grey and non-grey horse samples taken in pairs were evaluated using a Student’s *t*-test adapted to sample numbers below 30. A P-value <0.05 was considered as statistically significant (*).

### siRNA experiments

5 × 10^4^ of HoMel-A1 or HoMel-L1 cells were transfected with 50 pmol siRNAs (Ambion) using 5 μl LipofectamineTM 2000 (Invitrogen) in 1 ml Opti-MEM I (Invitrogen) per well in 12-well plates. The following siRNAs were used: pooled duplex 1 sense, 5′- GGAGCUCCUUCAUCUCCAAtt-3′ and duplex 2 sense, 5′- CGACUUCUGCCUUAAGUUUtt-3′ for *ARAF*; pooled duplex 1 sense, 5′-CCACAUCAUUGAGACCAAAtt-3′; duplex 2 sense, 5′-CAAUAGAACCUGUCAAUAUtt-3′ and duplex 3 sense, 5′-GGAAUCGAAUGAAAACUCUtt-3′ for *BRAF*; pooled duplex 1 sense, 5′-GGACUUUUCUUCAGAGAUAtt-3′; duplex 2 sense, 5′-GGACUGGAGUAAUAUCAGAtt-3′ and duplex 3 sense, 5′-CCAACACUCUCUACCGAAAtt-3′ for *CRAF*. The dinucleotide “tt” was added to all siRNAs to improve the stability after transfection. After 48 h, medium was changed to RPMI-1640 with 10% FBS. Quantitative PCR and Western blot were performed 72–96 h post-transfection. Biological triplicates were used for each siRNA treatment.

### Quantitative PCR

Total RNA was isolated from 1 × 10^6^ cells/ transfection using the RNeasy Mini Kit (Qiagen, CA) according to manufacturer’s protocol. The isolation included DNase treatment with the RNase-Free DNase Set (Qiagen, CA). 1 μg of total RNA was used to generate cDNA with the Advantage® RT-for-PCR Kit (Clontech Laboratories, Inc., Mountain View, CA, USA). SYBR Green quantitative PCR amplifications were performed on a Applied Biosystems 7900HT Sequence Detection System (Applied Biosystems, Life Technologies, Carlsbad, CA, USA). The primers used were ARAF_F 5′-CCGGCTCATCAAGGGGCGA-3′, ARAF_R 5′-GGACCCTGAGGGGTTAGCGG-3′, BRAF_F 5′-TGGATCCATTTTGTGGATGGCACC-3′, BRAF_R 5′-AGGGCTCTGATGCACTGCGG-3′, B2M_F 5′-GGCGGTTCTGAAAAACGAAAG-3′, B2M_R 5′-TCGAGCCTGACCAGAGCAT-3′, Eq_KRAS_F 5′-CATGAGGACTGGGGAGGGCTT-3′, Eq_KRAS_R 5′-AGCATCCTCCACTCTCTGTCTTGTC-3′, Eq_HRAS_F 5′-GACATCCACCAGTACAGGGAGCA-3′, Eq_HRAS_R 5′-CACCTCTGGGCCCTGCATCT-3′. The *CRAF* and *NRAS* primers were included in a ready-to-use mix from Qiagen, RT^2^ qPCR Primer Assay (Qiagen, CA). Reactions were carried out in a 10 μl volume containing 1X SYBR Green PCR Master Mix (Applied Biosystems, Life Technologies, Carlsbad, CA, USA), 0.7 μM of each primer and 3 μl cDNA. The thermal profile was 95°C for 10 min followed by 40 cycles of 95°C for 15 s and 60°C for 1 min. The relative mRNA expression levels were normalized to the endogenous housekeeping gene *B2M* and siNEG transfections for every cell line were used as a calibrator to evaluate the change in relative quantity.

### Statistical analyses

Statistical analyses were performed using the unpaired, two-tailed Student’s t-test.

## Results

### ERK1/2 activation in Grey horse melanomas

**Figure 1:**
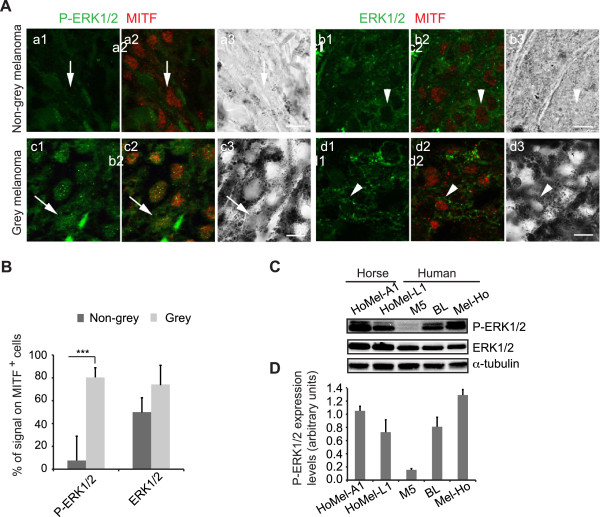
**Activation of the ERK pathway in melanoma of Grey horses. (A)** Double immunofluorescence staining was performed for P-ERK1/2 (green in a1-a2, c1-c2) or total ERK1/2 (green in b1-b2, d1-d2) and a nuclear melanocytic marker, MITF (red in a2, b2, c2, d2) in melanoma tissue sections from non-grey and Grey horses; a3, b3, c3, d3, corresponding bright field images of the sections. Arrows and arrowheads indicate representative cells for the corresponding stains. Scale bar, 10 μm. **(B)** Quantification of P-ERK1/2 and ERK1/2 immunofluorescent signals in relation to the total number of MITF^+^ cells in melanoma tissue sections from non-grey (n = 12; dark grey bars) and Grey (n = 19; light grey bars) horses shown as the mean ± s.e. of three independent experiments. ***P <0.001. **(C)** Western blot analysis of P-ERK1/2 levels in GHM *vs.* human melanoma cell lines with ^Q61R^RAS (BL), ^V600E^BRAF (Mel-Ho) and ^WT^RAS ^WT^BRAF (M5). **(D)** Quantification of total ERK1/2-normalized P-ERK1/2 protein levels in the cell lines listed in (C). The mean ± s.e. of three independent Western blots are shown.

Given the importance of the MAPK/ERK pathway activation in melanoma development, we examined the levels of the activated (phosphorylated) ERK1/2 (P-ERK1/2) in primary cutaneous melanoma tumours from Grey (n = 19) and non-Grey (n = 12) horses of different breeds from three geographic locations across Europe by indirect immunofluorescence, using an anti-MITF antibody to mark melanocytic lineage [23]. All the tumours expressed nuclear and occasionally cytoplasmic P-ERK1/2, however, both signals were by far more abundant in the GHM samples (80.2% ±8.5 *vs*. 7.6% ±21.3 in Grey and non-grey MITF^+^ cells, respectively; Figure 1A, B). Although non-grey melanomas were much more heterogeneous for the P-ERK1/2 staining than the Grey counterparts, the quantitative difference between the signals reached statistical significance (*P* <0.001). The total ERK1/2 signal was similarly heterogeneous in both Grey and non-grey samples (71.2 ± 17.4 *vs*. 50.5% ±13.0 in MITF^+^ cells of the respective melanoma type; Figure 1A, B). In line with the elevated P-ERK1/2 levels in the GHM tissues, high P-ERK1/2 levels were detected in two GHM cell lines, HoMel-L1 and HoMel-A1, established from a primary and metastatic melanoma tumour of a Grey Lipizzaner and Arabian horse, respectively [24]. The P-ERK1/2 levels were comparable to those seen in human melanoma cell lines with oncogenic BRAF or NRAS mutations, in contrast to a cell line with wild-type BRAF and NRAS (Figure 1C, D; Table 1). ERK1/2 was activated even in the absence of serum and serum addition had a minimal stimulatory effect on the P-ERK1/2 levels (Additional file 2: Figure S1A, B).

**Table 1:** **Genotypes of the melanoma cell lines used in this study**

	Melanoma cell line				
	Horse		Human		
***Gene***	HoMel-A1	HoMel-L1	BL	Mel-Ho	M5
*NRAS*	WT	WT	Q61R	WT	WT
*BRAF*	WT	WT	WT	V600E	WT

### MEK/ERK module is required for growth of Grey horse melanoma cells

**Figure 2:**
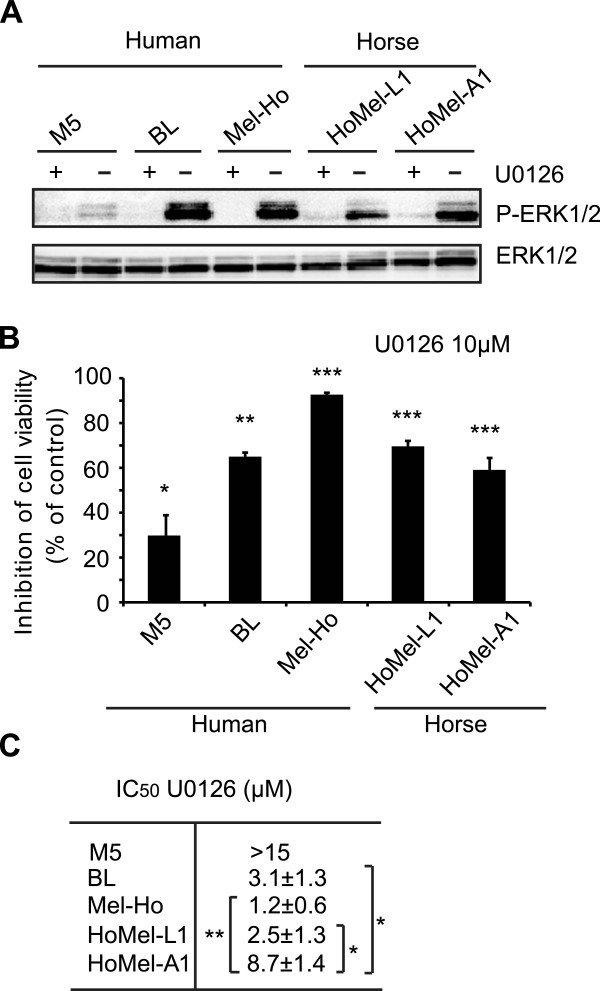
**The ERK pathway is growth-promoting in Grey horse melanoma cells. (A)** Grey horse and human melanoma cell lines were cultured in the presence of DMSO as vehicle control (-) or 10 μM MEK1/2 inhibitor U0126 (+) for 12 h following a 2 h serum-free preincubation. The effect of U0126 on ERK1/2 activation was analyzed by Western blot. Similar results were obtained in three independent experiments. **(B)** The effect of U0126 on cell growth was measured in relation to DMSO-treated control by Alamar Blue assay 72 h post-treatment. The data show the mean ± s.e. of a representative experiment of three independent experiments performed in triplicates. **(C)** The efficiency of U0126 treatment on cell growth was determined by calculating the concentration necessary to achieve a 50% reduction in cell proliferation (IC_50_). The data represent the mean ± s.e. of three independent experiments performed in triplicates. **(B, C)** *P <0.05; **P <0.01; ***P <0.001.

To assess the involvement of the ERK pathway in proliferation of the GHM cells, we treated the HoMel-L1 and HoMel-A1 cell lines with U0126, a specific inhibitor of MEK1/2 and therefore ERK phosphorylation. Western blot analysis showed an expected decrease in P-ERK1/2 in both cell lines upon the treatment (Figure 2A). The treatment also largely reduced cell viability in both cell lines (Figure 2B). As judged from the calculated IC_50_ values, HoMel-L1 appeared more sensitive to the MEK1/2 inhibition than HoMel-A1 (P <0.05) and at least as sensitive as the human ^Q61R^NRAS line BL (Figure 2C). Both GHM cell lines were less sensitive to the U0126 treatment than the human ^V600E^BRAF line Mel-Ho, although, this did not reach statistical significance for HoMel-L1. In contrast, cell viability of the human ^WT^BRAF ^WT^RAS line M5 was only weakly inhibited by the treatment (Figure 2B). These results demonstrate that ERK signaling is an important component for GHM cell growth; however the incomplete inhibition by U0126 suggests that additional ERK-independent mechanisms are involved.

### Activation of ERK pathway in Grey horse melanoma cells is not linked to common oncogenic alterations

To test whether the constitutive ERK1/2 activation was due to the presence of oncogenic mutations commonly associated with ERK1/2 activation in melanocytic neoplasms, we screened the horse cell lines and seven additional GHM tumours for mutations in exons 11 and 15 of *BRAF*, the full coding regions of *N*-, *K*- and *HRAS*, exon 4 and 5 of *GNAQ* and *GNA11*, and exons 9–21 of *KIT*. In all the genes except *KIT*, no mutations were found, indicating the wild-type status of the genes in this melanoma type. In *KIT*, four single nucleotide polymorphisms (SNPs) were found in exon 14, 15, 19 and 20 (11% each) in each of 4 different tumours (Table 2). However, these SNPs were silent on the protein level and present in the constitutional DNA from both Grey and non-Grey horses of different breeds (Table 2), and therefore were considered as common germline polymorphisms with no link to the Grey phenotype.

**Table 2:** ***KIT*** **polymorphisms in tumour DNA of Grey horses**

UCSC	Polymorphism (cDNA) ^a^	Exon	Seq	Protein	Study
chr3:77739479	c.2112A > G	14	TAA(A/G)AAC	silent	1
chr3:77737226	c.2181C > T	15	TGT(C/T)GTA	silent	2
chr3:77731814	c.2613C > T	19	AGT(C/T)GAT	silent	2
chr3:77731334	c.2739C > T	20	ATT(C/T)AAG	silent	1

^a^Numbering refers to accession number AF055037.

1 [25].

2 [26].

**Figure 3:**
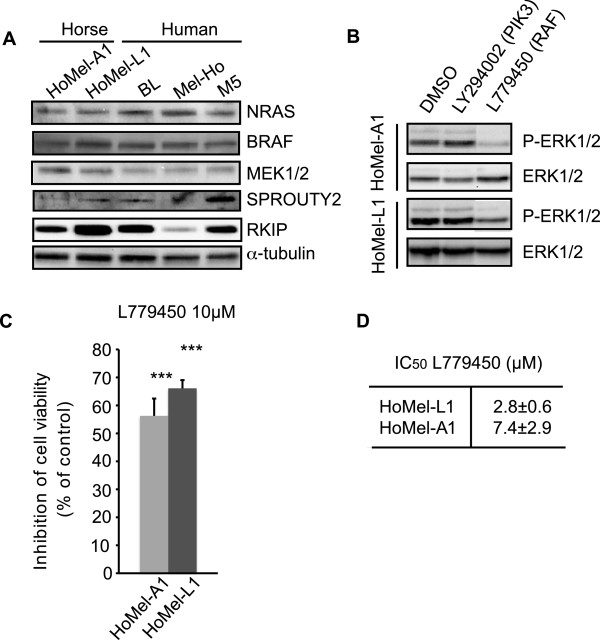
**The effects of upstream signaling pathways on ERK1/2 activation in Grey horse melanoma cells. (A)** Levels of NRAS, BRAF, MEK1/2, SPROUTY2 and RKIP expression in Grey horse *vs.* human melanoma cell lines analyzed by Western blot. **(B)** The effects of PI3 kinase/AKT and RAF pathways on ERK1/2 activation were analyzed using inhibitors LY294002 (50 μM) and L779450 (10 μM), respectively. The cell lines were cultured 19 in the presence of DMSO as vehicle control or with the indicated concentrations of the inhibitors for 12 h following a 2 h serum-free preincubation and their effect on ERK1/2 activation was analyzed by Western blot. Similar results were obtained in three independent experiments. **(C)** The effect of the RAF kinase inhibitor L779450 (10μΜ) on cell growth of GHM cell lines was measured in relation to DMSO-treated control by Alamar Blue assay 72 h post-treatment. The mean ± s.e. of three independent experiments performed in triplicates is presented. ***P <0.001. **(D)** The efficiency of L779450 (10μΜ) treatment on cell growth was determined by calculating the concentration necessary to achieve a 50% reduction in cell proliferation (IC_50_). The data represent the mean ± s.e. of three independent experiments performed in triplicates.

Another possible mechanism for a constitutive activation of the ERK pathway may involve overexpression of a component of the pathway or underexpression of its negative regulator. To address this possibility we compared expression levels of the key kinases and two major negative regulators of the pathway, SPROUTY2 and RKIP, in the GHM *vs*. human melanoma cell lines with activated ERK1/2 due to oncogenic BRAF or NRAS mutations. We found no substantial differences in the levels of ERK1/2 (Figure 1C), MEK1/2, BRAF, NRAS (Figure 3A and Additional file 3: Figure S2A) between the horse and human melanoma cells. The levels of SPROUTY2 and RKIP in the horse lines were not lower than those in the human lines (Figure 3A and Additional file 3: Figure S2A). Together, these results suggest that neither oncogenic mutations in the components of the ERK pathway nor alteration in their expression or of that of the pathway’s major negative regulators is likely to be responsible for the constitutive activation of ERK1/2 in GHM cells.

### ERK1/2 activation is BRAF, CRAF and KRAS-dependent in Grey horse melanoma cells

**Figure 4:**
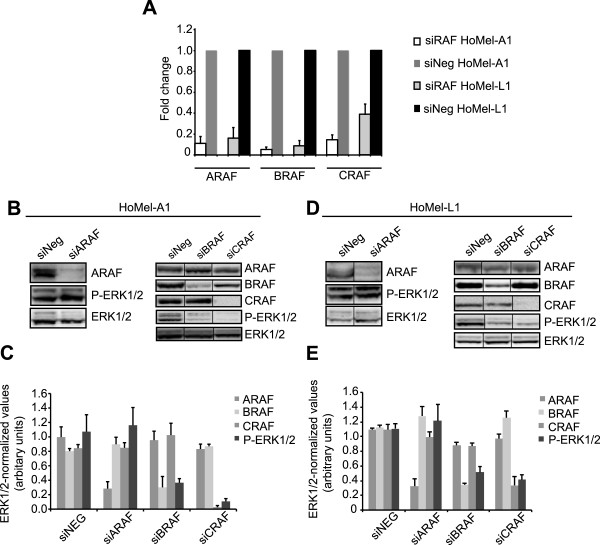
**ERK1/2 activation is B- and CRAF-dependent in GHM cells. (A)** Fold change of *ARAB*, *BRAF* and *CRAF* mRNA after silencing with specific siRNAs in HoMel-A1 and HoMel-L1 cells as compared to scrambled control siRNA (siNeg). Fold changes are expressed as a range, with the standard deviation of the ΔΔCT value incorporated into the fold change calculation. **(B, D)** Contribution of individual RAF kinases to ERK1/2 activation in HoMel-A1 and HoMel-L1 cells after silencing with specific siRNAs or scrambled control siRNA as assessed by Western blot analysis. Representative images of one of three independent experiments are shown. **(C, E)** Quantification of total ERK1/2-normalized ARAF, BRAF, CRAF and P-ERK1/2 protein levels in individual silencings. Shown are the mean ± s.e. of three independent experiments.

**Figure 5:**
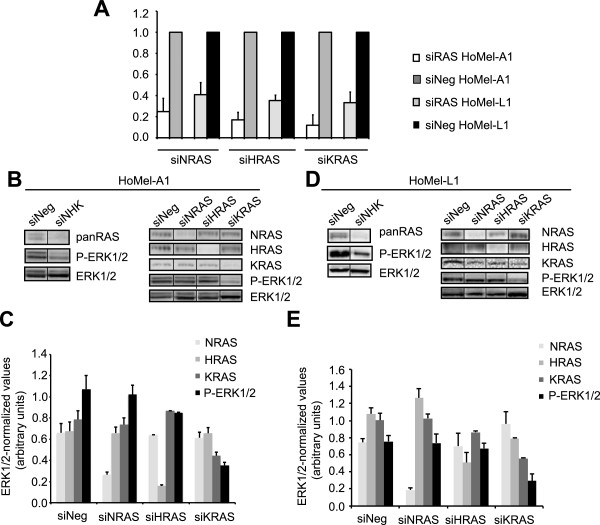
**ERK1/2 activation is mediated by KRAS in GHM cells. (A)** Fold change of *NRAS*, *HRAS* and *KRAS* mRNA after silencing with specific siRNAs in HoMel-A1 and HoMel-L1 cells in comparison to scrambled control siRNA (siNeg). Fold changes are expressed as in Figure 4A. **(B, D)** Combined (left panels) and individual (right panels) contribution of RAS kinases to ERK1/2 activation in HoMel-A1 and HoMel-L1 cells as assessed by Western blot analysis. Representative images of one of two independent experiments made in biological triplicates are shown. **(C, E)** Quantification of total ERK1/2-normalized NRAS, HRAS, KRAS and P-ERK1/2 protein levels in individual silencings. Shown are the mean ± s.e. of the two independent experiments.

In order to identify upstream signaling components involved in the constitutive ERK1/2 activation in GHM cells we used specific inhibitors against RAF (L779450), RAS (farnesyl thiosalicylic acid, FTS) and PI3K/AKT (LY294002) proteins. Only the L779450 treatment was able to reduce P-ERK1/2 levels, suggesting involvement of RAF kinases in the control of ERK activation (Figure 3B and Additional file 3: Figure S2B; FTS treatment not shown). The treatment attenuated cell growth in both cell lines (Figure 3C, D) to the levels comparable to those attained by the U0126 treatment, supporting a role of RAF kinases in GHM cell growth through ERK1/2 activation. We also examined the contribution of individual RAF isoforms on ERK1/2 activation by RNA silencing (siRNA). While no considerable reduction in ERK1/2 activation was achieved by ARAF depletion, BRAF and CRAF silencing each reduced the level of ERK1/2 phosphorylation (Figure 4A-E). Since RAS kinases are known upstream activators of wild-type RAF kinases, the failure of the FTS treatment to affect the P-ERK1/2 levels was somewhat unexpected. We therefore decided to directly manipulate the levels of RAS kinases by siRNA-based approach to address their potential contribution to the ERK1/2 pathway in GHM cells. The initial experiment with combined depletion of NRAS, HRAS and KRAS indicated their involvement in the signaling (Figure 5B, C left panels). Further investigation of individual contribution of the RAS isoforms indicated KRAS as major RAS activator of the ERK signaling in these cells (Figure 5B-E).

### ERK pathway is activated already in skin melanocytes of Grey horses

**Figure 6:**
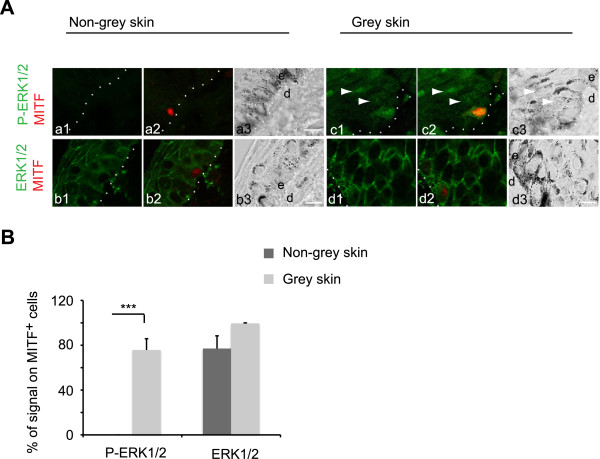
**Activation of ERK pathway in skin melanocytes of Grey horses. (A)** Double immunofluorescence staining was performed for P-ERK1/2 (green in a1-a2, c1-c2) or ERK1/2 (green in b1-b2, d1-d2) and MITF (red in a2, b2, c2, d2) in skin tissue sections from non-grey and Grey horses; a3, b3, c3, d3, bright field images of the sections. Dotted lines, skin basal membrane; e, epidermis; d, dermis. Scale bar, 10 μm. Note an increase in P-ERK1/2 signal (arrow heads in c1-c2) in the surrounding keratinocytes in Grey horse skin. **(B)** Quantification of P-ERK1/2 and ERK1/2 immunofluorescent signals in relation to the respective total number of MITF^+^ cells in skin tissue sections from non-grey (n =9; dark grey bars) and Grey (n =12; light grey bars) horses shown as the mean ± s.e. of three independent experiments. ***P <0.001.

The absence of the oncogenic mutations commonly linked to the activation of the ERK pathway in melanomas, the strong association of the *Grey* mutation with the melanoma predisposition [17] and the notion that the *Grey* mutation has an effect throughout melanocyte development [18], prompted us to test if the ERK pathway was already activated at the level of normal skin melanocytes in Grey horses. We analyzed expression of both phosphorylated and total ERK1/2 in skin samples from Grey (*n* =9) *vs*. non-grey horses (*n* =12) of different breeds from the same and different geographic locations, as was done for the melanoma samples. A high percentage of MITF^+^ epidermal melanocytes positive for the P-ERK1/2 was detected in all Grey horse skins (75.8% ±10.0) in sharp contrast to non-grey horse skins, where the phosphorylated ERK was absent (Figure 6A, B). The activated ERK1/2 was detected both in the cytoplasm and the nucleus in Grey melanocytes. Percentage of the total ERK1/2 positive cells was 99.5% ±0.5 in Grey and 77.1% ±11.3 in non-grey skins (Figure 6A, B). Interestingly, keratinocytes from Grey but not non-grey horses were also positive for P-ERK1/2 (Figure 6A). The staining pattern of both P-ERK1/2 and ERK1/2 was highly reproducible in samples coming from different studs and prepared in different laboratories.

## Discussion

Constitutive activation of the ERK pathway is present in the overwhelming majority of melanocytic tumours characterized to date and has been assigned a pivotal role in melanomagenesis. Distinct oncogenic aberrations in the components of the pathway or in the upstream signaling cascades have been linked to activation of the pathway in different melanocytic neoplasms. In this study, we detected activated ERK1/2 in 100% of the examined cutaneous melanomas and cell lines of Grey horses as well as in cutaneous melanomas of non-grey horses, thus recapitulating the universal importance of this pathway in melanomagenesis. The levels of the activated ERK1/2 were significantly higher in the Grey horse samples, most likely reflecting a difference in the underlying molecular phenotype and/or melanoma stage. In contrast to the majority of human melanocytic neoplasms, where activation of ERK is linked to the presence of somatic activating mutations in either *RAS*, *BRAF*, *GNAQ*/*GNA11* or *KIT*, these mutations were not found in our GHM samples. The ERK activation was neither linked to changes in the expression of main components of the pathway (*i.e*. NRAS, BRAF, MEK1/2 and ERK1/2) nor its major negative regulators (*i.e.* SPROUTY2 and RKIP). Pharmacological inhibition of the MEK/ERK module in GHM cell lines demonstrated that this pathway provides a growth-promoting signal in these cells. The signal was found to be mediated by both BRAF and CRAF kinases as demonstrated by pharmacological inhibition and siRNA-assisted depletion of the proteins. In melanomas harboring activated BRAF, activation of MEK/ERK is achieved by this isoform [27], while in melanomas with oncogenic RAS, the activating signal to ERK is passed by the ^WT^CRAF, due to deregulation of its inhibition [28]. In melanomas with ^WT^NRAS and ^WT^BRAF proteins, ERK activation is usually achieved via ^WT^BRAF from the activating upstream signals [29]. The involvement of both ^WT^BRAF and ^WT^CRAF kinases in the ERK signaling has been previously observed in human melanocytes [30], but is rather unusual in a melanoma context. Depletion of individual RAS isoforms by specific siRNAs identified KRAS as another upstream activator of the ERK pathway, with the signal proceeding most likely via RAF kinases. Further studies are needed to find out whether KRAS is involved in the activation of both BRAF and CRAF isoforms as well as to identify the upstream activating signaling component(s).

As we have shown previously, the *Grey* mutation is the primary cause of the Grey horse phenotypes including melanoma [17]. Furthermore, experiments using transgenic zebrafish suggested that the *Grey* mutation is active throughout melanocyte development [18]. These notions, combined with the absence of commonly found ERK-activating mutations, prompted us to examine if the *Grey*-associated activation of the ERK pathway was already present at the level of skin melanocytes. We indeed found high levels of P-ERK1/2 in all epidermal melanocytes examined regardless of horse age, in sharp contrast to the non-grey counterparts. While normal skin melanocytes do not show measurable amounts of P-ERK, its expression increases as melanocytes undergo neoplastic transformation [31, 32]. The elevated levels of activated ERK1/2 in normal skin melanocytes in Grey horses (even before the melanoma onset) therefore suggest their general predisposition to melanoma genesis. The notion that only a portion of melanocytes with activated ERK will develop melanoma, suggests that the ERK activation is an initial event in GHM genesis and additional alterations are needed for progression to melanoma. Grey horse melanomas are always dermal, however, evidence for their origin in dermal melanocytes is missing and migrating epidermal melanocytes has been suggested as a source of the tumours [16]. Our observation of the ERK activation in epidermal melanocytes supports the latter hypothesis, although we have not performed a thorough analysis of the P-ERK1/2 expression in the dermal melanocytes. Interestingly, we also observed ERK activation in the surrounding keratinocytes. Further studies are necessary to clarify the melanocyte-keratinocyte interactions in Grey horses.

## Conclusions

This study demonstrates that the 4.6 kb duplication in *STX17* in Grey horses is a novel mutation associated with constitutive activation of the ERK pathway in melanocytic cells. We have recently reported the presence of a higher copy number of the *Grey* mutation in more aggressive Grey horse melanomas [19], suggesting that the duplicated sequence may constitute a melanoma-driving element. Further studies are underway to provide evidence for the direct mechanistic link of the *STX17* duplication to the ERK pathway activation and melanoma development.

The present study also shows that somatic activating *BRAF*, *RAS*, *GNAQ*, *GNA11* and *KIT* mutations, frequently observed in human melanomas, are not required for melanoma development in Grey horses. The constitutive ERK activation in Grey horse melanoma therefore strengthens it as a model for the human counterparts where mutations with similar to the *Grey* mutation’s effects may be contributing to melanoma development particularly in the cases lacking common somatic oncogenic mutations.

## Electronic supplementary material

Additional file 1: **Supplementary Methods.** (DOC 28 KB)

Additional file 2: Figure S1: Effect of serum addition on ERK1/2 activation in GHM cells. (A) After overnight serum starvation, HoMel-A1 (A) and HoMel-L1 (L) cells were serum-stimulated for the indicated time periods and analyzed by Western blot for P-ERK1/2 and ERK1/2. (B) Quantification of total ERK1/2-normalized P-ERK1/2 protein levels shown as the mean ± s.e. of three independent Western blots. (PDF 314 KB)

Additional file 3: Figure S2: Supplimentary data to Figure 3A and B. (A) Quantification of tubulin-normalized NRAS, BRAF, MEK1/2, RKIP and SPROUTY2 protein levels in the horse and human melanoma cell lines shown as the mean ± s.e. of three independent Western blots. (B) Quantification of total-ERK1/2-normalized P-ERK1/2 levels in HoMel-A1 and HoMel-L1 cells treated with inhibitors for PI3 kinase/AKT (LY294002, 50 μM) and RAF kinases (L779450, 10 μM) expressed as % of DMSO control. The cell lines were cultured in the presence of DMSO as vehicle control or with the indicated concentrations of the inhibitors for 12 h following a 2 h serum-free preincubation and their effect on ERK1/2 activation was analyzed by Western blot. The mean ± s.e. of three independent Western blots are shown. (PDF 75 KB)
