# Supplementary material for: Constitutive activation of the ERK pathway in melanoma and skin melanocytes in Grey horses
Source: BMC Cancer. 2014 Nov 21;14:857. doi: 10.1186/1471-2407-14-857 (PMC4254013; doi:10.1186/1471-2407-14-857)
Supplement: Supplementary file 1 — Additional file 1: Supplementary Methods. (DOC 28 KB) [file 12885_2013_5052_MOESM1_ESM.doc]

*Analysis of BRAF, RAS, GNAQ, GNA11 and KIT mutations*

The following primers were used to obtain the horse amplicons:

*BRAF* exon 11 forward, 5′-TCCCTTTCAGGCATAGGGTA-3′ and reverse, 5′-TGACATGTGACAAGGTCATTGTAT-3′;

*BRAF* exon 15 forward, 5′-TCATAATGCTTGCTCTGATAAGAAA-3′ and reverse, 5′-CAGCATCTCAGGGTCCAAA-3′;

*NRAS* exon 1 and 2 forward, 5′-CACTAGCACCTAGCGCTTTCA-3′ and reverse, 5′-CAGCGAAAGGGTATGGGTAA-3′;

*NRAS* exon 3 forward, 5′-CATTGCATTCCCTGTGGTTT-3′ and reverse, 5′-CACACTTACAGATCATCCTTTCAGA-3′;

*NRAS* exon 4 forward, 5′-CCTTGCTGTTTATCCCTTCC-3′ and reverse, 5′-GCCAGATGGTGAAGGCTGTA-3′;

*NRAS* exon 5 and 6 forward, 5′-TGAGGGAGAGCTTACAACTTGG-3′and reverse, 5′-GAATTTGCGTAAGTGGCACA-3′;

*HRAS* exon 1 and 2 forward, 5′-GGGACAGGAGACCACTGAAG-3′and reverse,

5′-AGATGCAGATGGAGGACAGG-3′;

*KRAS* exon 1 forward, 5′-CATGTTCTAATTGAGTCACATTTTCA-3′ and reverse, 5′-AAGCATGAGCCTGCACAAAT-3′;

*KRAS* exon 2 and 3 forward, 5′-CCAGACTGTGTTTCTCCCTTC-3′ and reverse, 5′-CAATTACTCCTCCATGTCAATTT-3′.

*GNAQ* exon 5 forward, 5′-ATTAATATGAATATTGTTAACCTTGCA-3′ and reverse, 5′-GAGAGCACTCACCTCATTGTC-3′.

*GNA11* exon 4 forward, 5′-CGCCCGTCCCACTGTGT-3′ and reverse, 5′- CCCGAGGAAGCCACGC-3′;

*GNA11* exon 5 forward, 5′-CAGCACTGATCCCCGGC-3′ and reverse, 5′- CTGCCCCAGGCCCTCA-3′.

*KIT* exon 9 forward, 5′-TGCTTTGTACATCCTCTTGC-3′ and reverse, 5′-GTGCATGGACAGAACACAC-3′;

*KIT* exon 10 and 11 forward, 5′-GCTGTGAGATGGGAGGTG-3′ and reverse, 5′-CAAAGCTATCAAAGGTGGTG-3′;

*KIT* exon 12 and 13 forward, 5′-CACCACCTTTGATAGCTTTG-3′ and reverse, 5′-TTTGGATGCAACATACGTG-3′;

*KIT* exon 14 forward, 5′-CGGGTTTTGATAAGCAATG-3′ and reverse, 5′-CCACGATGAGAGAAACAGG-3′;

*KIT* exon 15 forward, 5′-AAAGGCCATCTAGCTCCTG-3′ and reverse, 5′-CAACCCTTAACTGCCATTG-3′;

*KIT* exon 16 forward, 5′-TTCACGTAGGGTCTCATGG-3′ and reverse, 5′-CCAAAGAGACAGCAGTTGG-3′;

*KIT* exon 17 forward, 5′-TGGAGTTGGTTTTGAAAGTG-3′ and reverse, 5′-ACGTGCCCCATAATTACAC-3′;

*KIT* exon 18 and 19 forward, 5′-TCATCTGAGGCCATACAGG-3′ and reverse, 5′-AAGGGCCATTTAGCATCAC-3′;

*KIT* exon 20 forward, 5′-AAGGGCCAAGATGTGTTTC-3′ and reverse, 5′-GCCCAATTTATAGCCCAAG-3′;

*KIT* exon 21 forward, 5′-GCTGTTGGGTTGTAGTTGG-3′ and reverse, 5′-CAACCATCCTTTTGGACAG-3′.

PCR was performed using a touch-down program (annealing at 63 to 53°C for 7 cycles followed by annealing at 56 or 59°C for 37 cycles) in 25 μl reactions using 50 ng DNA, 1M betaine (Sigma Aldrich, St Louis, MO, USA) and 0.6U AmpliTaq Gold (Applied Biosystems, Foster City, CA, USA). The products were sequenced with ABI3730XL sequencer (Applied Biosystems, Foster City, CA, USA) and analyzed for mutations with CodonCode Aligner (CodonCode Corporation, Dedham, MA, USA).
