# Supplementary figures and images for: Constitutive activation of the ERK pathway in melanoma and skin melanocytes in Grey horses
Source: BMC Cancer. 2014 Nov 21;14:857. doi: 10.1186/1471-2407-14-857 (PMC4254013; doi:10.1186/1471-2407-14-857)

**A**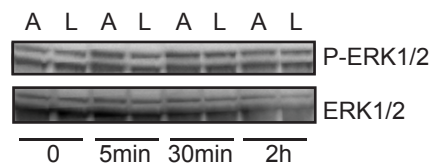**B**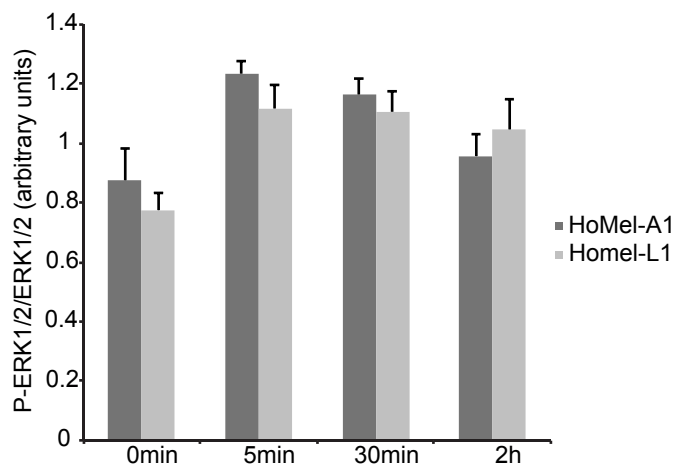

Supplement: Supplementary file 2 — Additional file 2: Figure S1: Effect of serum addition on ERK1/2 activation in GHM cells. (A) After overnight serum starvation, HoMel-A1 (A) and HoMel-L1 (L) cells were serum-stimulated for the indicated time periods and analyzed by Western blot for P-ERK1/2 and ERK1/2. (B) Quantification of total ERK1/2-normalized P-ERK1/2 protein levels shown as the mean ± s.e. of three independent Western blots. (PDF 314 KB) [file 12885_2013_5052_MOESM2_ESM.pdf]

**A**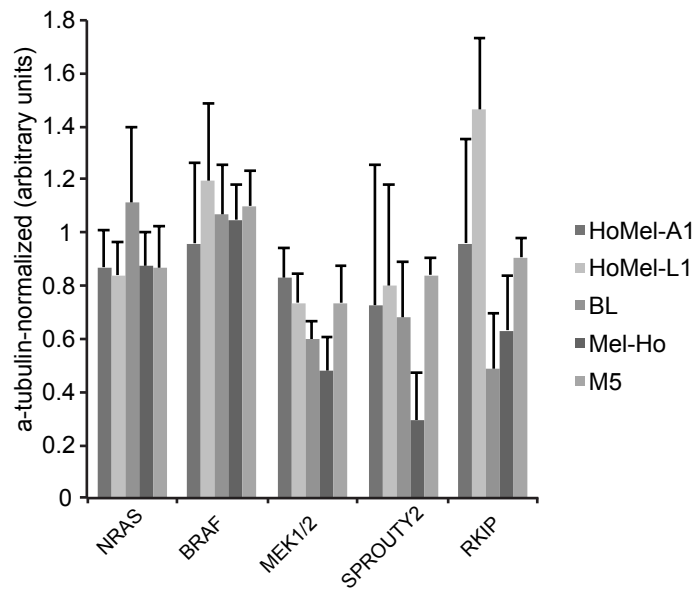**B**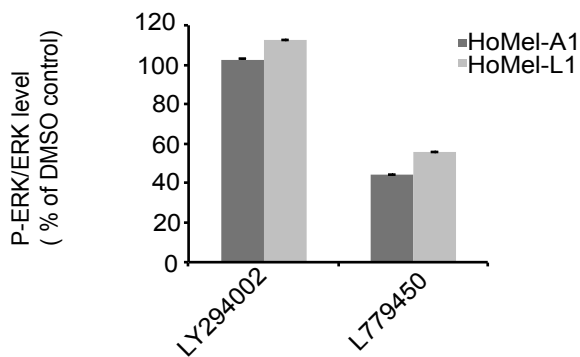

Supplement: Supplementary file 3 — Additional file 3: Figure S2: Supplimentary data to Figure 3A and B. (A) Quantification of tubulin-normalized NRAS, BRAF, MEK1/2, RKIP and SPROUTY2 protein levels in the horse and human melanoma cell lines shown as the mean ± s.e. of three independent Western blots. (B) Quantification of total-ERK1/2-normalized P-ERK1/2 levels in HoMel-A1 and HoMel-L1 cells treated with inhibitors for PI3 kinase/AKT (LY294002, 50 μM) and RAF kinases (L779450, 10 μM) expressed as % of DMSO control. The cell lines were cultured in the presence of DMSO as vehicle control or with the indicated concentrations of the inhibitors for 12 h following a 2 h serum-free preincubation and their effect on ERK1/2 activation was analyzed by Western blot. The mean ± s.e. of three independent Western blots are shown. (PDF 75 KB) [file 12885_2013_5052_MOESM3_ESM.pdf]
